# Sustainable Transportation Attitudes and Health Behavior Change: Evaluation of a Brief Stage-Targeted Video Intervention

**DOI:** 10.3390/ijerph15010150

**Published:** 2018-01-18

**Authors:** Norbert Mundorf, Colleen A. Redding, Andrea L. Paiva

**Affiliations:** 1 Department of Communication Studies, Harrington School of Communication and Media, University of Rhode Island, Kingston, RI 02881, USA; 2 Cancer Prevention Research Center, Department of Psychology, College of Health Sciences, University of Rhode Island, Kingston, RI 02881, USA; credding@uri.edu (C.A.R.); apaiva@uri.edu (A.L.P.)

**Keywords:** sustainable transportation, active living, transportation and health, Transtheoretical Model of Change, environmental health

## Abstract

Promoting physical activity and sustainable transportation is essential in the face of rising health care costs, obesity rates, and other public health threats resulting from lack of physical activity. Targeted communications can encourage distinct population segments to adopt active and sustainable transportation modes. Our work is designed to promote the health, social, and environmental benefits of sustainable/active transportation (ST) using the Transtheoretical Model of Change (TTM), which has been successfully applied to a range of health, and more recently, sustainability behaviors. Earlier, measurement development confirmed both the structure of ST pros and cons and efficacy measures as well as the relationship between these constructs and ST stages of change, replicating results found for many other behaviors. The present paper discusses a brief pre-post video pilot intervention study designed for precontemplators and contemplators (N = 604) that was well received, effective in moving respondents towards increased readiness for ST behavior change, and improving some ST attitudes, significantly reducing the cons of ST. This research program shows that a brief stage-targeted behavior change video can increase readiness and reduce the cons for healthy transportation choices.

## 1. Introduction

Sustainable, or active transportation (ST), defined as commuting by any means other than a single occupancy vehicle (SOV), represents one effective way to increase physical activity, reduce emissions and related threats, and improve quality of life across populations [1,2,3]. The dependence on SOV transportation has been increasing since the end of World War II. Obvious benefits from increased mobility have come with the significant drawbacks of SOV transportation, including: pollution, congestion, injuries and fatalities, and unfortunate urban land-use choices with suburban sprawl [4]. In addition, the public health community is increasingly concerned about the impact of sedentary lifestyles and poor energy balance resulting from the reliance on SOV transportation, recommending population-based strategies to increase physical activity levels [5]. Despite the many synergistic benefits of sustainable transportation, the vast majority of Americans commute alone by car or truck. Awareness of the health and environmental impacts of SOV driving is lacking as many drivers observe this behavior to be the social norm—which has been widely promoted by corporations and institutions for decades. A vital part of both our culture and economy, it is often not even perceived to be a choice. As part of the shift towards a healthier and more sustainable lifestyle, we need to find ways to make changes in transportation choices more urgent by emphasizing local, current, and tangible threats, while simultaneously providing options that are both sustainable and feasible for individuals. 

### 1.1. Transportation, Health and Environment 

Mixed-use urban design, population density, connectivity, access to walkways, parks and bikeways have been implemented to encourage walking and biking. Cultural and social factors are also associated with active transportation modes. Many of these factors are long-term and difficult to change. Communities, state and federal agencies have implemented measures that can increase active transportation and improve population health. Transportation choices also have important socio-economic influences. Reliable and efficient transportation is a key ingredient of economic success and social mobility; safe, efficient, and appealing public transit, as well as safe biking and walking alternatives are critical for the health and wellbeing of children, families, commuters, and elderly community members.

Clearly, given the prevailing urban and suburban sprawl, most U.S. residents will not be able to commute to work, school or other locations by walking or biking alone. However, research has shown that public transit users tend to walk significantly more than automobile commuters [6] with potentially significant health benefits. Also, Morency, Demers and Polinquin [7] estimated that converting short motorized trips to walking would allow 8.3% of their study population to increase physical activity levels, potentially improving fitness and weight management. One American Public Health Association study [8] concluded that the near-complete dependence on automobile travel results in further costs for road construction and repair, continued urban sprawl and reduced walkability, less physical activity, health problems due to sedentary lifestyle, pollution and car crashes, and enormous long-term direct and indirect costs. 

Economy, environment, and health can all benefit greatly from increasing sustainable transportation (ST). Well-designed transportation systems can provide exercise opportunities, improve safety, lower emotional stress, link economically disadvantaged people to opportunity, connect isolated older adults and people with disabilities to crucial services and social support, and stimulate economic development. Conventional automobile-focused planning often overlooks the health impacts of transportation investments.

The heavy commuter reliance on SOV is also one of the most preventable contributors to the carbon footprint of campuses and communities [9]. In addition to structural and technical innovations, behavior change is pivotal to reducing SOV travel. The current study is part of our research program designed to apply the Transtheoretical Model of Change (TTM) to different populations to improve transportation choices, the environment, and quality of life in campus communities. Given the success of TTM in other areas of behavior change (see below), our team has developed a series of innovative projects, which can serve as models for increasing sustainable transportation for campuses and communities nationwide.

### 1.2. Population Health 

Transportation and the built environment have gained public attention as key factors for the quality of life, health, environment, and social coherence of a community. In particular, the dominance of the automobile has been associated with negative impacts on community life [10]. Communities, urban planners, researchers and artists are seeking more sustainable solutions. Key challenges are to raise awareness, launch a lively public discourse, propose and visualize solutions.

Walking and biking have attracted research attention as healthy transportation alternatives (within reasonable distances), or as part of an intermodal transportation model [11]. A significant number of people walk to work, school, or to shop (those who live within an acceptable distance with a safe route); many more walk for recreation. Unlike much of Europe, China and many developing countries, biking in the U.S. tends to be mainly for recreation [12]. One detailed interview study concluded that biking is somewhat popular among American elementary school children, but they lose their interest once they enter middle school [13]. Few U.S. high school students (mostly males) bike for fun or transportation.

### 1.3. Public Transit, Walking and Biking

Transit is most available and most used by commuters in urban areas. The mode split is different in urban compared to suburban and rural areas. Urban transit is far more available, and in safe urban areas, walking is a good transportation option. Walking and biking are the primary healthy transportation activities and contributors to active living. Researchers have investigated motivations for biking [12], where safety is a key consideration—both traffic and personal safety. Pedestrians and bikers are victimized at higher rates (relative to their mode share) compared to automobile occupants. This is the case for traffic injuries and fatalities, and also for criminal victimization. While traffic accidents are far more common than criminal incidents involving pedestrians or bike riders, the latter may be more visible and can reduce the perceived safety of a neighborhood, bike path, or park [11].

Sheepers and colleagues [14] analyzed various incentives designed to promote active transportation, physical activity and to reduce negative impacts of SOV transportation. Their findings are encouraging in that almost all published research studies in their analysis, found positive effects on (sustainable) mode shift from car use to active transportation. They categorized intervention tools as legal, economic, communicative (media, behavioral) and physical (e.g., bike rentals, improved facilities) and found that typically more than one intervention tool was used, combining social marketing, individualized transportation plans, improved facilities or financial incentives.

Brög and colleagues [15] were instrumental in developing and promoting Individualized Travel Marketing, which uses a number of ‘soft’ measures along with travel planning tools to encourage sustainable transportation, specifically public transit use. Their analysis of German commuters found that 81% relied on single occupancy driving. Further analysis showed that while sustainable transportation may be currently impractical for about 56%, the remaining 25% of SOV commuters have a clear choice with reasonable alternatives. In addition, another 32% could be reached with reasonable improvements in infrastructure, etc. In order to encourage people who have a reasonable choice of transportation alternatives, informational and attitudinal interventions were recommended. Brög et al. reported success on three continents with large scale interventions; their interventions were particularly effective in promoting the use of sustainable transportation following structural improvements (e.g., a new train line).

A number of researchers have focused on the Theory of Planned Behavior (TPB), Norm Activation, and related theories to explore variables impacting transportation choice [16,17]. Lauper and colleagues [18] investigated TPB variables and compared behavioral intentions and self-reported travel with more “objective” GPS-based travel records, which generally found a weaker relationship between intention and actual behavior. Their interesting and informative structural equation model combined attitudes and behavioral intentions with habit and cost related to sustainable transportation. The study also highlighted the need to address a combination of psychological and external factors. In other words, investments in infrastructure and financial incentives are important, but to be effective they have to be supported by communication and behavior change strategies. A recent meta-analysis of 58 primary studies assessed determinants of travel mode choice, including both behavioral intentions and actual behaviors [19]; it found that intentions, habits and past use were the strongest predictors, followed by other TPB constructs. Environmental attitudes played a role in shaping behavioral intentions, but their effect on actual behaviors was negligible.

### 1.4. Transtheoretical Model

The Transtheoretical Model of Change (TTM) addresses the behavior-intention gap by gradually moving people from indifference or ignorance towards increased readiness and finally to action in a series of descriptive and prescriptive stages of change (see below) [20]. TTM can be used to target stage-specific strategies to effect individual behavior change and engage entire communities in sustainable transportation choices and policy initiatives. For sustainable transportation in the U.S. and other countries with high reliance on SOV transportation, the vast majority of commuters and travelers are in the earliest stages of change (precontemplation and contemplation, see below). A key TTM finding over the past 30 years has been that moving people forward by one stage will roughly double the odds that they will ultimately take action and change their behavior [21]. This paper presents a simple low-cost option specifically useful for moving those at the earliest stages.

The Transtheoretical Model (TTM) has been recognized as a leading approach to changing health behaviors. TTM has been successfully applied to more than 50 health behaviors [22], including smoking, diet, and exercise [20,22]. TTM interventions have been successful at moving entire populations, including people who are not interested in changing, towards change and in encouraging people to sustain behavior changes in the long-term [20,23,24]. Smoking cessation is the most widely studied behavior change using the TTM, with measurement development research leading to tailored intervention development and randomized trials evaluating the efficacy of TTM interventions [25]. Importantly, TTM intervention efficacy for smoking cessation has also been replicated and extended in new populations and with multiple behavioral targets, including some studies by independent investigators [26,27,28,29,30]. This program of research for smoking cessation and other health behaviors provides direction and promise for TTM research applied to sustainable transportation [31]. In order to compare instruments and interventions to promote change in transportation behavior, TTM was used to compare transportation choices and behaviors at two New England state universities with different transportation infrastructure and travel patterns [32].

One central construct of the TTM is the stage of change. Longitudinal studies have found that people move through a series of five stages when modifying behavior on their own or with the help of formal interventions [20,31,32]. In precontemplation (PC), individuals may deny a problem and/or be resistant to change; they may be unaware of the negative consequences of their behavior, or may have given up on change because they are demoralized. They are not intending to change in the foreseeable future. Individuals in contemplation (C) are more likely to recognize the benefits of changing. However, they continue to overestimate the costs of changing and, therefore, are ambivalent and not yet ready. Individuals in preparation (PR) have decided to change soon, and have begun to take small steps toward that goal. People in action (A) are overtly engaged in modifying their behavior and are working to prevent relapse. Those in maintenance (M) have sustained change for at least six months and may not need to work as hard to prevent relapse as their behavior change becomes more habitual [20]. The TTM improves the likelihood of behavior changes by tailoring or targeting interventions to each individual’s stage of change. The TTM also includes constructs, decisional balance and self-efficacy that have demonstrated systematic relationships to the stages of change [22,31,32]. Decisional balance assesses individuals’ evaluations of the benefits and costs of changing, and/or the cost savings of adopting a new behavior. These TTM constructs have been validated in the area of sustainable transportation [31,32,33]. Meta-analyses across many randomized trials, including a range of different health behaviors, have found that TTM tailored interventions were more effective than non-tailored interventions [23,24,31,32].

One effective TTM-based intervention study increased active commuting among U.K. employees. Mutrie and colleagues demonstrated that a TTM-based self-help intervention effectively helped employees who were in either the contemplation or preparation stage to initiate active commuting (walking or cycling) to work [34]. Two Australian studies also used TTM to show how to reduce SOV use as a primary transportation mode. Shannon and colleagues [35] assessed potential for change as well as barriers and motivators affecting the transportation choices of 1040 students and 1170 staff in Perth at the University of Western Australia. A strong relationship between stages of change for adopting active transportation (walking, biking, public transit use) and pros and cons of change and self-efficacy (confidence in using active modes) was found, confirming TTM predictions. Attitude and behavior patterns were more favorable compared to the U.S., but they also showed potential for reaching out to those not yet engaged in active transportation. Rose used *TravelSmart* software to target 2977 new Monash University students to increase the use of ST modes and reduce SOV travel [36]. Students received individually tailored travel information, as well as various incentives. A single tailored intervention produced progress for those at each stage of change over the course of the school year [36].

This work is part of an interdisciplinary project designed to promote ST. Supported in part by the University of Rhode Island Transportation Center, this work lays part of the foundation for developing effective interventions to promote ST behavior change, including everyone, even those who may not currently be ready for such a change. 

### 1.5. ST Interventions

The assessment of ST behaviors provided empirical support for developing transportation interventions to promote ST, both within and beyond the campus setting. An important finding from the measurement development study was that the TTM model applied to ST, just as it had been successfully applied to numerous health and other behaviors [31,32]. Also, and importantly, patterns of cross-sectional relationships between pros and cons, efficacy, and stages of change for ST were comparable to the patterns that were found for other health behaviors [31,32]. A study of faculty, staff and students at two public universities in the Northeast was designed to integrate the TTM applied to transportation behavior with geospatial modeling to evaluate impacts on students and older University commuters [32]. Once implemented and demonstrated as effective, TTM interventions could be scaled to apply to a range of diverse commuters beyond campus environments.

## 2. Materials and Methods

A brief multimedia video intervention was designed and evaluated as one way to better inform future behavior change campaigns. The study was approved by the Institutional Review Board at the University of Rhode Island for the protection of human subjects (IRB code 203219-3).

### 2.1. Measures

#### 2.1.1. Demographic and Travel Items

A series of single items assessed participant characteristics including gender, racial/ethnic group, university affiliation, access to a car, bike, et cetera. Participants rated their future likelihood of using various sustainable transportation modes.

#### 2.1.2. Stages of Change for Sustainable Transportation (ST)

Stages of change for ST was assessed both pre-test and post-test using the following item (without stage labels) based on prior research [31]: “Sustainable transportation includes any way of getting to [school] other than driving by yourself (single occupancy vehicle use). So walking, biking, public transportation (bus/subway/train) and carpooling are all means of Sustainable Transportation”. Then, participants chose one statement from the following options:

(1) I do not regularly use ST and I do not intend to start within the next six months (PC);
(2) I am thinking about using ST regularly within the next six months (C);
(3) I plan to use ST regularly within the next 30 days (PR);
(4) I use ST regularly and have been for less than six months (A); or
(5) I use ST regularly and have for six months or more (M).

#### 2.1.3. Decisional Balance for Sustainable Transportation

This scale was administered both pre-test and post-test. Decisional balance assessed pros and cons, had good measurement structure, and replicated previously established relationships with stages of change in college students, staff and faculty [31]. The pros (5-item *α* = 0.84) and cons (5-item *α* = 0.77) demonstrated good internal consistencies. Individuals rated the importance of various ST benefits (pros) and costs (cons) in their own decision making, including such potential benefits as, saving money, being green, and improving their own and the planet’s health. Cons items reflected potential barriers such as time, practicality, and difficulty. Higher scores reflect stronger endorsement of scales.

#### 2.1.4. Self-Efficacy for Sustainable Transportation

This scale was administered both pre-test and post-test. This 5-item self-efficacy scale (α = 0.82) also had good measurement properties and replicated hypothesized relationships with stages of change in college students, staff and faculty [31]. Participants rated their confidence that they would use ST, even when faced with challenges, such as when it was inconvenient, or they were tired, or running late. Higher scores reflect higher levels of confidence in ability to use ST in spite of challenges.

#### 2.1.5. Evaluation Items

Four items were administered after participants viewed the video intervention, at post-test only. Participants rated their level of agreement on a four-point scale with each item.

### 2.2. Procedures

Participant volunteers completed the study entirely online. Some undergraduate students received partial credit in a course in exchange for study participation. After informed consent, a pre-test survey was followed by a link to the 4-min intervention video, followed by the post-test survey. The video was designed to increase the pros of ST and to show transit users, bike riders, and walkers, emphasizing the relative ease of using ST to promote self-efficacy.

The one-time, brief video intervention was developed primarily targeting early stage (PC and C) college students. This video primarily utilized appealing video of transit riders getting on the bus at an urban terminal, walkers, and bicyclists on campus, along with brief interview clips focusing on the health benefits of ST. The 4-min video primarily showed a diverse range of students, and a few participants were older, to include staff and faculty. In keeping with the TTM findings that interventions for precontemplators and contemplators should primarily focus on the pros (benefits) of change, the interviews and video clips emphasized positive aspects of using ST, especially getting physical activity, improving health, saving money, and relaxing, listening to music, or getting work done on the commute. These pros were underscored by an overall positive mood and the beautiful weather on a sunny spring day. The video also emphasized efficacy in that peers were shown walking, biking, and riding the bus. At the time of writing this, the video was available on YouTube: https://www.youtube.com/watch?v=0xjyhTr1dQM.

## 3. Results

N = 604 participants were in the PC and C stages and were included in this study (see Table 1). Table 1 describes pre-test participant characteristics. About two-thirds of the participants (66.4%) were students, with about 25% faculty and staff. The sample’s mean age was 28.3 years (SD = 13.9). Most participants were female (63.3%) and white (91.7%). Pre-test ST stages of change reflected that most participants (79.9%) were not thinking about using ST within the next six months (PC), and some (20.1%) were considering using ST within the next six months (C). Participants rated the likelihood of using four ways to increase their own future ST behaviors: carpooling (51%) was rated most likely, followed by walking (13.2%), public transportation (10.9%), and biking (8.8%).

Of these, N = 527 (87.3%) viewed the video and completed the post-test evaluation questions. Table 2 describes both PC and C stage group responses to post-test evaluation questions and stage progress. Most respondents agreed or strongly agreed (82–84%) that they liked the program. They also agreed that the program gave them new things to think about (74–81%), could help them make some positive changes (78–81%), and increased their interest in ST (68–75%). Table 2 also shows that 38.7% of pre-test precontemplators and 16.2% of pre-test contemplators reported progress of at least one stage at post-test after viewing the video.

Small attitude changes from pre- to post-testing were also examined: a nonsignificant trend towards an increase in mean ST pros (*t*(472) = 1.905, *p* < 0.057) and a significant decrease in mean ST cons (*t*(462) = 2.504, *p* < 0.013) was found. No difference between pre-test and post-test ST confidence was found (*t*(475) = −1.559, *p* < 0.120).

## 4. Discussion

This brief, stage-targeted video intervention was able to improve some ST attitudes, specifically, reducing the cons of ST, and increase readiness in precontemplators and contemplators recruited from a university setting. These results can help transportation and public health professionals change commuter behavior at a time of limited resources and increase interest in mode change to reduce traffic, conserve fuel, and promote active, sustainable transportation. This research demonstrates that the TTM is a useful tool for encouraging ST behavior change and healthy transportation choices. Even a brief video targeted at those who did not yet use ST was able to decrease the cons of ST and increase the likelihood of future ST use. Given the brief video and the inclusion of only early stage participants, this study’s failure to find changes in ST confidence at post-test was not surprising. However, one goal of this video was to increase the pros of ST and although there was a nonsignificant trend found, this video did not achieve this aim. TTM measures [31] were not only useful in and of themselves, but they provided an empirical basis for development of this brief film and can provide a foundation to develop and evaluate future computer-tailored interventions (CTI) that could demonstrate value both for research and practice [23,24].

This study is one part of a program of research that supports the application of the TTM to sustainability-related behaviors and the ability of brief targeted behavioral interventions to increase people’s willingness to engage in sustainable behaviors and climate resilience [37]. TTM measures can provide the empirical foundation for TTM intervention development research applied to additional sustainable behaviors (e.g., recycling, green eating [38], energy conservation, land/water resource management, climate adaptation and resilience [37]). To achieve the goal of active transportation as part of a more sustainable society, communications designed to promote individual behavior change are critical. Not only do individual behavior changes impact sustainability directly, TTM research has found that such change is often associated with policy support as well [39]. In addition, those in the action and maintenance stages of sustainable behavior can be role models for others, thus providing social support and normative support for more widespread changes. Also, we expect that this approach can be applied to other kinds of sustainable behaviors in the future. TTM research has shown that even very different health and environmental behaviors can be evaluated and intervened upon using common constructs. This innovative approach has the potential to reach diverse population segments and to help provide tools for lasting change.

Attitude and behavior change regarding mobility options will gain future importance both for individuals and policy makers. Individuals will face increased gasoline prices, more congestion and pollution, and more negative consequences of sedentary lifestyles. Policy makers face these same congestion and pollution concerns, along with limited resources for new road and highway construction. The social and public health costs of sedentary lifestyles will increase the sense of urgency and hopefully attention, to sustainable transportation behaviors. Finally, the pressure to mitigate the rate of climate change and to be prepared for its future impacts necessitates a reduced dependence on fossil fuel based transportation. Individual consumer choices along with policy decisions will both be needed to facilitate the necessary changes. The TTM has provided a roadmap for change, and these results can play an important, even if small, part. Since targeted multimedia messages and Computer Tailored Interventions are easily scaleable, they have strong potential to reach large demographic and geographic segments at relatively low cost.

One important dimension not addressed in this research is access to healthy and sustainable food choices, also known as green eating [38]. While overweightness and obesity are growing challenges, some trends are encouraging. Smaller stores (bodegas) have begun to stock fruits and vegetables more visibly. Farmer’s markets and community gardens are increasingly popular. Smaller stores and some farmer’s markers can be located within walking distances, while larger grocery stores can be reached by major bus lines. Improving access to healthy food for those who already walk and use public transit can better address growing rates of obesity and metabolic syndrome [2,3,5]. Sustainable transportation and access to healthy and local food are key ingredients to improving the quality of urban life [11].

This study has some important limitations. A convenience sample was recruited from one university setting and is unlikely to fully represent U.S. commuters. A pre-post design with no control group was used to examine attitudes and readiness for ST. Future research should replicate and extend these findings with more representative, diverse, and generalizable samples, especially from community and urban settings. Future randomized trials that include a control group could provide stronger evidence of the effects on not only attitudes and readiness, but ST behaviors as well. Support for ST policy and infrastructure was not examined in this study and should be included in future studies.

## 5. Conclusions

Promoting physical activity and access to community resources is critical in the face of rising healthcare costs, childhood obesity and many public health threats resulting from insufficient physical activity. The built environment, transit, walkability, food access, overall population health and quality of life are interconnected. Transportation options can allow individuals to utilize job and educational opportunities that can be economic engines for sustainable growth, especially important for disadvantaged population segments, minorities, the disabled, elderly, and urban youth. Promoting transportation alternatives can promote social justice, especially in areas with limited access to automobiles. Transit provides access to work, school, employment, grocery stores, farmers markets, health care, and social activities. Public transit also promotes walking (and some biking) since most riders walk to and from transit stops to their destinations. Clearly, pedestrian traffic safety and neighborhood safety are important components supporting both active transportation and community vitality.

The transportation culture needs to change. The primacy of the automobile has been pervasive for too long and for most of us it appears to be the only reasonable means of transport. By strengthening multimodal thinking (and options), we can simultaneously reduce the negatives of extensive automobile traffic and our dependence on automobiles. Our program of research reflects a low-cost approach to changing transportation attitudes and behaviors; it is scaleable and lends itself to integration with individualized travel information and/or tailored feedback, which is increasingly available online via computers and smart phones. Future research should build upon these findings to develop larger scale, more effective population-based sustainability interventions. The Transtheoretical Model can serve as a framework to improve healthy and sustainable behaviors, including, but not limited to transportation. Building upon and extending this research can contribute to efforts to meet synergistic public health, community resilience, and sustainability goals.

## Figures and Tables

**Table 1:** Demographic information.

Demographics	N	%
**Gender**		
Female	362	59.9
Male	209	34.6
not reported	33	5.5
**Racial/Ethnic Group**		
Asian/Hawaiian/Pacific Islander	9	1.5
Black	17	2.8
Hispanic	13	2.2
White	523	86.6
Other	9	1.5
not reported	33	5.5
**University Affiliation**		
Student	401	66.4
Faculty	65	10.8
Staff	90	14.9
Both student & staff/faculty	24	4.0
Neither Student nor staff/faculty	24	4.0
**Pre-test ST Stage**		
Precontemplation	478	79.1
Contemplation	126	20.9
**Access to Car**		
I own my own vehicle	535	88.6
I share a Vehicle	27	4.5
Neither own nor share	40	6.6
not reported	2	0.3
**Own a Bicycle**		
Yes	119	19.7
No	443	73.3
not reported	42	7.0
	**Mean**	**SD**
**Age**	28.3	13.9

**Table 2:** Post-test agreement with evaluation items and stage progress.

	PC	C
Evaluation Items—% Agree/Strongly Agree	n = 416	n = 111
I liked this program.	83.7	81.7
This program could help me make some positive changes.	73.7	80.7
This program gave me some new things to think about.	78.0	80.6
This program increased my interest in ST.	67.7	75
Stage Progress		
% moved forwards at least one stage	38.7	16.2

Note: N = 523–527 for each item due to missing item level data.
